# Asexual reproduction and growth rate: independent and plastic life history traits in *Neurospora crassa*

**DOI:** 10.1038/s41396-018-0294-7

**Published:** 2018-11-09

**Authors:** Jennifer L. Anderson, Bart P. S. Nieuwenhuis, Hanna Johannesson

**Affiliations:** 1 Department of Organismal Biology, Uppsala University, Norbyvägen 18D, SE-752 36 Uppsala, Sweden; 2 Department of Ecology and Genetics, Uppsala University, Norbyvägen 18D, SE-752 36 Uppsala, Sweden; 3 Division of Evolutionary Biology, Faculty of Biology, Ludwig-Maximilians-Universität München, Grosshaderner Strasse 2, Planegg-Martinsried, 82152 München, Germany

**Keywords:** Evolution, Fungal biology

## Abstract

Trade-offs among traits influencing fitness are predicted by life history theory because resources allocated to one function are unavailable to another. Here we examine the relationship between two such traits, asexual reproduction and growth rate, in the filamentous fungus *Neurospora crassa*, where shared genetic and physiological factors and a source–sink energetic relationship between growth and reproduction may constrain the evolution of these traits. To test growth–reproduction relationships in this species, we independently selected on mycelial growth rate or asexual spore production in a heterogeneous lab-derived population and evaluated the response of the non-selected traits. Combined with phenotypes for the 20 wild strains used to produce the heterogeneous population and the genome-wide genotypes of 468 strains, these data show that growth and reproduction are highly plastic in *N. crassa* and do not trade off either among wild strains or after laboratory selection in two environments. Rather, we find no predictable growth–reproduction relationship in the environments tested, indicating an effective absence of genetic constraint between these traits. Our results suggest that growth rate and asexual reproduction may not respond predictably to environmental change and suggest that reliance on a single trait as a proxy for fitness in fungal studies may be inadvisable.

## Introduction

Fitness, the ability of an organism to survive and reproduce in its environment and thereby transmit its genes to future generations [1], is determined by myriad interacting, and potentially environmentally sensitive, traits. Balance among investments in these traits is expected to optimize fitness according to the theory of life history evolution, such that beneficial changes in traits related to survival or reproduction are achieved at the cost of other life history traits. These trade-offs may result from the discrete partitioning of a limiting resource, physiological limitations, or the antagonistic action of underlying genes [2–4]. However, pairs of life history traits can be more or less genetically integrated depending on their underlying genetic architecture [5]. Pleiotropy among life history traits can be positive or negative, either facilitating or constraining their evolution [5]. Further, the expression of individual life history traits and the relationships between them can differ by environment [6, 7] to the extent that phenotypic and genetic correlations between traits can change in both sign and magnitude under different conditions [8]. Even the genetic basis of a trade-off may differ between environments [9]. Thus, life history traits may not trade off as predicted by theory (e.g., ref. [10]) and relationships between them can differ between environments.

Growth and reproduction are iconic performance traits in life history evolution theory [2]. Trade-offs between components of these traits (e.g., rates of each, size at maturity, timing of investment) have been reported in plants [11], vertebrates [12, 13], and some fungi, such as the coprophilous basidiomycete fungus *Coprinus cinereus* [14]. However, growth and reproduction are positively associated in the white button mushroom *Agaricus bisporus* [15] and two ascomycete species of *Aspergillus* [16, 17], while there is no association between growth and fruiting in the split gill mushroom *Schizophyllum commune* [18]. Understanding relationships between growth and reproduction in filamentous fungi is important for understanding fungal evolution [19] and ecology (e.g., competition [16, 17]), and predicting impacts of environmental change [20]. However, growth–reproduction relationships and their environmental dependencies are largely unknown for fungi. Here, we capitalize on knowledge and resources for the fungus *Neurospora crassa* to dissect relationships between growth rate (GR) and asexual reproduction in two laboratory environments.

Growth in *N. crassa* is achieved by the apical extension and branching of hyphae (filaments) throughout a substrate from which nutrients and energy are obtained via absorptive nutrition and transported throughout the fungus. Asexual spores (macroconidia; hereafter referred to as spores) are produced on a specialized subset of hyphae (conidiophores) that grow out of the substrate. Spores in *N. crassa* can serve as nuclear donors in sexual reproduction or develop into independent colonies. During growth and the colonization of new substrates, GR is expected to positively influence competitive ability, and thus survival, by increasing resource availability [16, 17]. Overall, reproduction is a resource sink dependent on growth, and growth is both a source and a sink [21]. Optimal investment in each is expected to be highly environment-dependent [19, 22]. Based on resource partitioning, it is difficult to predict how changes in either GR or reproduction in *N. crassa* would impact the other. However, predicted genetic integration between growth and reproduction suggests a positive relationship between these traits. During colony development, reproductive regions and the actively growing substrate hyphae at the colony edge have been found to share expression profiles for 11% (492 genes) of the more than 4000 genes studied [23]. This genetic integration may in part reflect the shared physiological process of hyphal extension [23].

**Fig. 1:**
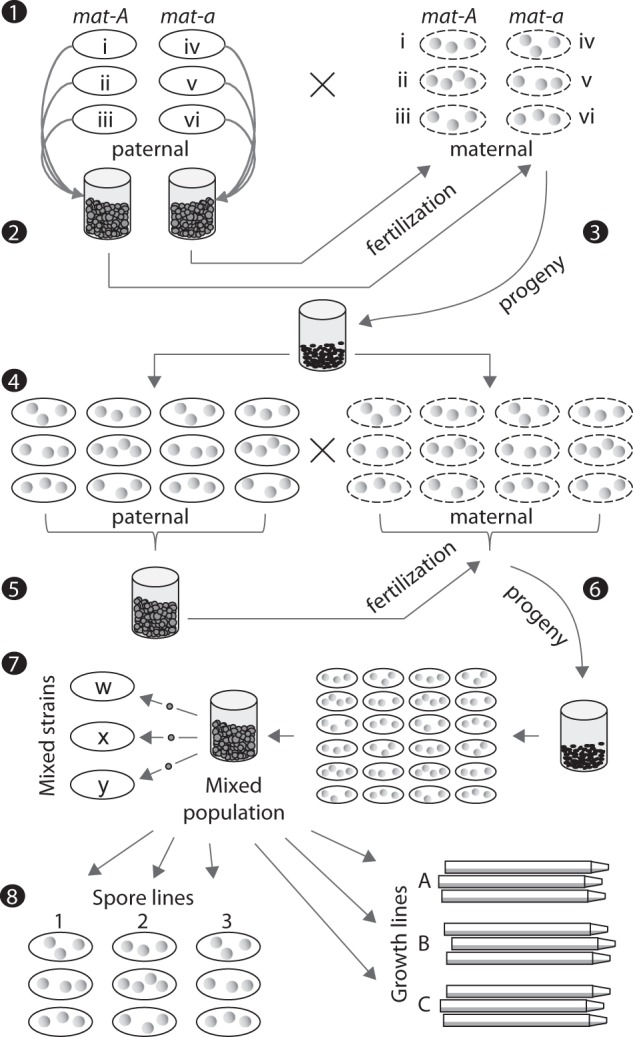
Production of the lab-derived “mixed” population. (1) All 20 wild strains (for simplicity only six are depicted (i–vi)) were cultured under “paternal” and “maternal” conditions (solid and dashed oval “Petri dishes”). Colonial growth on SGF is shown as gray circles. (2) Spores of like mating type were pooled and used to fertilize wild strains of the opposite type. (3) The progeny (ascospores) from all successful crosses were pooled and (4) cultured both under paternal and maternal conditions. (5) Spores from all paternal plates were harvested, pooled, and used to fertilize colonies on all maternal plates. (6) The resulting progeny were pooled and grown at a large population size under paternal conditions. Spores from these plates were harvested and frozen. This spore pool constitutes the mixed population. (7) Mixed strains are strains of single spore origin from the mixed population. (8) Growth and spore selection lines each originated from a sub-sample of the mixed population. See text and SI Methods for the actual numbers and identities of wild strains used, and numbers of spores and colonies at each step. Diagram features not to scale

Whereas life history evolution theory classically predicts a trade-off between growth and reproduction, in *N. crassa*, shared genes and physiology and the source–sink energetic relationship between growth and reproduction may otherwise constrain the independent evolution of these traits. Thus, the questions remain: how integrated are growth and reproduction in *N. crassa*, and how sensitive are these traits and the relationship between them to environment? To answer these questions, we combined existing strain and methodological resources for this genetic model species with the robust experimental selection framework of life history evolution. Using 20 wild strains, we generated a heterogeneous lab-derived population (mixed population; Fig. 1). We separately selected for increases in GR and asexual spore production (reproductive output (RO)) in this mixed population and evaluated correlated responses to selection in the non-selected phenotype. To gain further insights into this otherwise “black box” process, we used genome-wide genotyping to track the origins and distributions of diversity from wild strains, through the mixed population, to the selected lines. Both evidence from wild strains and from complementary selection experiments support the conclusion that these highly plastic life history phenotypes are largely independent in *N. crassa*.

## Materials and methods

### Strains and culture environments

Twenty wild strains of *N. crassa* from the distinct Louisiana and Caribbean populations [24] (10 of each mating type; SI Methods) and two *fl* tester strains of known mating type (FGSC #4317 and #4347) were obtained from the Fungal Genetics Stock Center (Manhattan, KS, USA; SI Methods) [25]. Sterile techniques were employed throughout.

We used two main culture environments; Vogel’s medium [26] (1.5% agar) augmented with either 1% w/v sucrose (hereafter referred to as “sucrose”) or a combination of 1% l-sorbose, 0.05% glucose, and 0.05% fructose (hereafter referred to as “SGF”). Sucrose medium supports robust growth and abundant asexual reproduction, whereas growth on SGF is highly branching, and was used to grow this fungus in a compact colonial form with approximately 100 colonies per 9 cm Petri dish. A low nitrogen version of SGF was used to induce fruiting for “maternal” cultures in sexual generations (below, SI Methods). Strains were grown at 25°C with 12 h light/dark cycles in 9 cm Petri dishes unless noted.

### Mixed population

To study segregating variation in new genomic contexts, a heterogeneous lab-derived population (hereafter referred to as “mixed population”) was produced via two generations of random mating among the 20 wild strains (Fig. 1, full details in SI Methods). Briefly, each wild strain was grown to produce protoperithecia (“maternal” reproductive structures) and separately to produce asexual spores (spores herein). Spores from the wild strains were pooled by mating type in equal numbers, spread over each maternal wild strain culture, and thus used as “paternal” units, nuclear donors, in fertilization. Progeny (sexually produced ascospores) resulting from successful crosses in the first generation were used to establish the next generation of “maternal” and “paternal” cultures, 5000 and 3000 ascospores, respectively. Spores from all “paternal” cultures were pooled and used to fertilize the “maternal” plates. Progeny from this second generation of crosses (5000 ascospores) were grown to produce spores which were pooled, frozen at −80°C [27], and constitute the “mixed population.” Individual spores isolated from a thawed sample of the mixed population and grown independently are hereafter called mixed strains. Prior to selection experiments, a sample of the mixed population was thawed (2500 spores) and cultured under standard conditions on SGF for 2 weeks. Spores produced on the thaw plates were pooled, stored at 4 °C, and used to initiate spore and growth selections (below).

### Spore selection

To select for high spore production, we sequentially transferred spores produced by independent sub-samples of the mixed population (lines 1, 2, and 3) on SGF, thereby numerically favoring genotypes producing more spores. Each line was established from approximately 2000 spores (across 20 plates with SGF). For each asexual generation, spores were washed from all plates in each line, pooled, filtered (to minimize inclusion of hyphal fragments), and again, a random sample of 2000 spores was used to initiate the next generation for a total of six generations (SI Methods). Spores from each line were frozen at the termination of selection. One hundred spores from the last generation of each line were isolated (Spore strains) and independently cultured from revived samples of each line.

### Growth selection

To select for fast linear growth, independent sub-samples (lines A, B, and C) from the mixed population were competitively grown and the fastest-growing mycelium used to establish the next generation. Growth selection occurred in race tubes, serological pipettes modified for horizontal unidirectional hyphal growth [28] (SI Methods), on sucrose. Approximately 6200 spores from the mixed population were used to initiate growth selection (across 62 race tubes, randomized into three lines). Cultures were grown in the dark for 4 days (3 days in generations 6–10), and then stored at 4 °C. To establish the next generation, agar containing the terminal growth was removed from all tubes in each line, blended with water containing Tween-20 (0.01%), condensed to a dense slurry, and used as inoculum for the next generation (250 µl slurry per tube). After generation 10, colonized agar fragments (~1 mm^2^) were isolated from the slurry and used to establish 100 independent cultures from each line (growth strains).

### Phenotypes

To measure RO, culture tubes with SGF or sucrose were inoculated with 50,000 spores per replicate (*N* = 3) per strain, grown for 1 week under standard conditions, and stored at 4 °C in the dark prior to harvesting spores using a standardized process (SI Methods). The number of spores produced per culture was calculated for each replicate based on cell counts, sample volumes, and dilution factors for cells in the size range of 4–10 μm on an Innovatis CASY DT cell counter (Roche, Penzberg, Germany; see also Supplemental Data). GR on sucrose was measured in race tubes in triplicate for each strain. Each replicate was inoculated with 50,000 spores and growth marked at ~24 h intervals post inoculation. Average GR (mm/h) was calculated from growth between 48 and 72 h, using the actual elapsed time interval. Because SGF restricts growth, GRs on SGF were estimated as radial growth in Petri dishes on cultures initiated with 5000 spores. Radial growth was marked in perpendicular axes marked at ~24 h intervals for mixed strains, and after 24 h and 9 days for the wild strains. Average radial GRs (mm/h) based on growth from 48 to 72 h or over 192 h (wild strains) were used to describe growth (SI Methods). Cultures were exposed to light during marking and after inoculation. GR and RO were measured for growth-selected and mixed strains on sucrose (growth + mixed), and for spore-selected and mixed strains on SGF (spore + mixed) in batches over 2 weeks each assay (SI Methods). Each batch contained approximately equal numbers of selected and mixed strains. Note that spores produced from sucrose cultures were used for all wild strain assays; all other strains were cultured on the test media prior to assay. Mating types were determined by crossing each strain with *N. crassa* fluffy mutants of known mating type.

Phenotypic variation among the wild strains and in the mixed population was assessed separately for each phenotype and environment using analysis of variance (ANOVA) with strain as the main effect (wild strains), or with batch as a main effect and strain as a nested effect (mixed population). Differences in the wild strains across environments were analyzed using ANOVA with effects strain, environment, and their interaction. Correlations between average phenotypes were tested using parametric and non-parametric approaches, Pearson's product–moment correlations, and Spearman’s *ρ*. Because wild strains grew slower on SGF than sucrose, GR was estimated over different timescales in the two environments and min–max normalized data were used for comparisons between environments. Clone correction was performed on the mixed population by treating each clone (below) as one group (SI Methods). To test responses to selection, we used ANOVA with main effect batch, line as a random effect, and strain as a random effect nested in line. Growth + mixed and spore + mixed assays were analyzed separately. Tukey’s honest significant difference was used to compare among the mixed population and selected lines, and least-square means contrasts were used to test the hypothesis that the selected lines differ overall from the mixed population. Analyses were performed in JMP v11 (SAS Institute Inc., Cary, NC, USA1989–2007).

### Genotypes

Genome-wide sequence polymorphisms were identified using Restriction site Associated DNA sequencing (RADseq). RADseq libraries were prepared using *Pst*I [29–31] (SI Methods), and sequenced at the SNP&SEQ Technology Platform (SciLifeLab, Uppsala, Sweden) on an Illumina HiSeq2500 (125 bp reads, v4 sequencing chemistry). Sequences were aligned to the *N. crassa* OR74A NC12 reference genome [32, 33], and variants called in Stacks v1.34 [34]. Identical genotypes (clones), and pairwise genetic distances were estimated using GenoDive v2.0b27 [35]. Polymorphisms that uniquely identify wild strains (IDtags) were identified (SI Methods) using a conservative dataset of 8555 RADtags for which all strains in the experiment were genotyped at all loci.

## Results

### Substantial phenotypic variation among wild and mixed strains

**Fig. 2:**
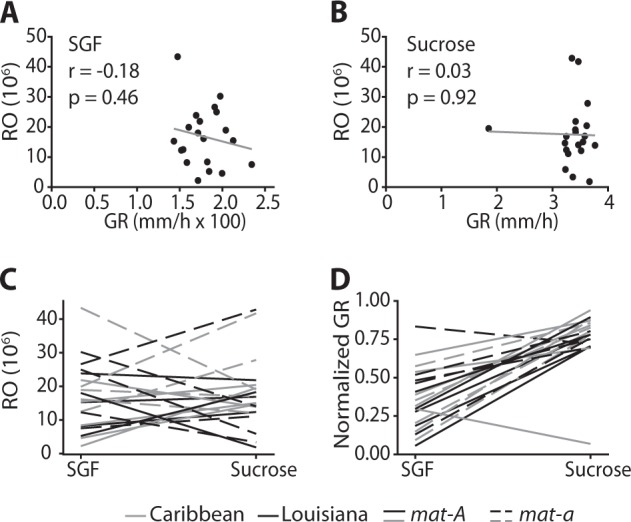
Growth rate (GR) and reproductive output (RO) in *N. crassa* wild strains. These traits are not correlated either when grown on SGF (**a**) or sucrose (**b**) and are highly plastic (**c**, **d**). We see no clear differences in phenotypes based on origin or mating type. Values are means (*N* = 3). Values for growth in **d** are min–max normalized, due to a differences in rate measurement on SGF and sucrose

GR and RO vary substantially in the wild and mixed strains (Figs. S1 and S2). Wild strains varied in both GR and RO in both environments (*N* = 20; sucrose GR: *F*_19_ = 13.58, *p* *<* 0.0001; RO *F*_19_ = 9.45, *p* < 0.0001; SGF GR: *F*_19_ = 8.96, *p* *<* 0.0001; RO *F*_19_ = 5.02, *p* < 0.0001), after prior cultivation on sucrose. Overall RO did not differ between environments (substrate: *F*_1_ = 0.32, *p* = 0.57) with an average of ~17 million spores produced per strain, but wild strains grew >100× slower in the SGF than sucrose environment (substrate: *F*_1_ = 457.51, *p* < 0.0001; Fig. 2 and S1). Although variation among strains for GR on sucrose was subtle, it was statistically significant even after removal of the slowest growing strain (4713) from analysis (strain: *F*_18_ = 2.30, *p* = 0.02). Among mixed strains, variation in GR was statistically significant in both environments in spite of batch effects (sucrose *N* = 105: batch *F*_3_ = 6.12, *p* < 0.001, strain *F*_104_ = 1.62, *p* = 0.002; SGF *N* = 92: batch *F*_3_ = 81.70, *p* < 0.0001, strain *F*_91_ = 15.80, *p* < 0.0001). In the mixed population, however, variation in RO among strains was statistically significant only on SGF (sucrose *N* = 49: batch *F*_1_ = 6.76, *p* = 0.01, strain *F*_50_ = 1.08, *p* = 0.38; SGF *N* = 45: batch *F*_1_ = 1.92, *p* = 0.17, strain *F*_44_ = 5.46, *p* < 0.0001). Adding clone as a random factor did not qualitatively affect these results.

### GR and reproduction are not correlated in the *N. crassa* wild and mixed strains

A classic approach for evaluating relationships between traits in life history evolution relies on the estimation of phenotypic correlations. We found no significant relationship between RO and GR among the 20 wild strains either on SGF (*r* = −0.18, *p* = 0.46) or sucrose (*r* = −0.02, *p* = 0.92). The absence of significant correlations in both environments persisted when analyzed using a non-parametric approach, which is less sensitive to extreme values (SGF: *ρ* = −0.06, *p* = 0.80; sucrose: *ρ* = 0.06, *p* = 0.81), and after removal of strains with extreme values (without 4715 on SGF *r* = 0.03, *p* = 0.92; and 4713 on sucrose *r* = 0.04, *p* = 0.87). Results from the mixed population supported the absence of this trade-off in *N. crassa* (Fig. S3), both with and without clone correction. Thus, evidence from phenotypic correlations between GR and RO suggests that these traits do not trade off in *N. crassa* in either environment. We note that phenotypic correlation approaches can be misleading in terms of their underlying genetic and physiological factors [36, 37], and further test this relationship via laboratory selection.

### Substantial phenotypic plasticity among wild and mixed strains

RO and GR are highly plastic in both the wild strains and mixed strains. Among the wild strains, variation in both RO and GR is environment-dependent (strain by substrate interaction: GR *F*_19_ = 9.92, *p* *<* 0.0001; RO *F*_19_ = 5.46 *p* < 0.0001). Plasticity, evident in plots of the reaction norms for each trait in the two environments for the wild strains (Fig. 2c, d), was qualitatively similar to that observed in the mixed strains (Fig. S3). Genotype-by-environment interactions, indicated by the crossing of reaction norms and resulting in rank changes, were found for both phenotypes. A priori, we expected both RO and GR to be reduced on SGF relative to sucrose due to the effect of sorbose on the growth pattern of *Neurospora*. However, in several strains RO was higher on SGF than sucrose when grown under otherwise identical conditions with the same available surface area (Fig. 2c).

### Genotypic variation in the mixed population

**Fig. 3:**
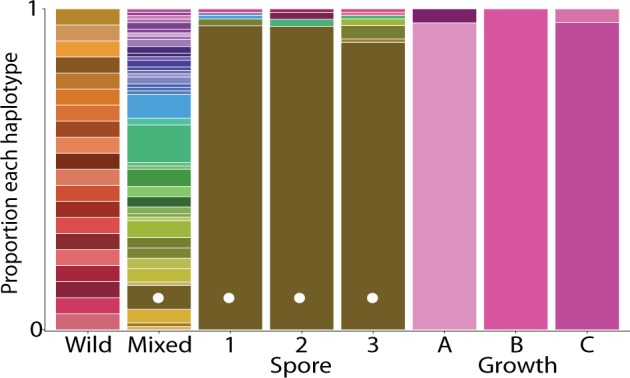
The relative frequencies of unique genome-wide haplotypes, based on 12,657 SNPs from 3466 RADtags. As indicated by unique colors, the 20 wild strains differed from each other, and from all other sampled strains. Forty-one clones were identified in the mixed population (*N* = 94). One of these clones (indicated by a white dot) was the dominant clone in all spore populations (*N*_1_ = 95, *N*_2_ = 92, *N*_3_ = 96). Unique clones, not sampled in the mixed population, dominated each of the growth populations (*N*_A_ = 23, *N*_B_ = 24, *N*_C_ = 24)

By mating individual strains from the mixed population to tester strains of known mating type, we determined that the mixed population was ~60% *mat*-a. Further, we genotyped the wild strains (*N* = 20), mixed strains (*N* = 94), spore strains (*N* = 283), and growth strains (*N* = 71) at loci associated with *Pst*I restriction sites to characterize genotypic diversity. We sequenced 25,094 RADtags covering ~14% of the genome (NCBI Sequence Read Archive accession SRP094745). A subset of 12,657 single-nucleotide polymorphisms (SNPs) from 3466 RADtags, genotyped in all strains with no ambiguous base or genotype calls, was used to classify genome-wide haplotypes. The wild strains were distinct from each other and all other strains, indicating effective recombination in the generation of the mixed population (Fig. 3). We identified 41 unique genome-wide haplotypes (unique clones; Fig. 3) in our sample of mixed strains. This result is robust and decreases only to 39 identifiable clones at a distance threshold of 1000 pairwise differences. The most abundant clone was sampled 11 times. Results specific to the selected lines are presented in context below.

**Fig. 4:**
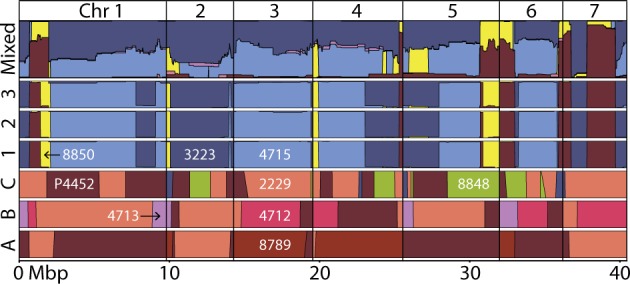
Genome-wide plot of the wild strain origins of genomic content in the mixed and selected populations based on allele frequencies of IDtags. DNA from eight wild strains was found in the mixed strains (see Fig. S4 for content from wild strains 8874, 3200, and 3199, which were sampled at low frequency). Diversity in the spore strains (1, 2, and 3) came from only four wild strains, whereas nine wild strains were represented in the growth strains (**a**–**c**). Frequencies for each population range from 0 to 1

We found 6423 IDtags across the genome with no missing data, 10 or fewer SNPs per tag, and no ambiguous base calls at polymorphic sites (SI Methods). These tags uniquely identify genomic content originating from each of the wild strains. Given the genome size of *N. crassa* (41.10 Mbp on seven chromosomes, ploidy = 1*n*), this translates to approximately one IDtag each 7227 bp. Using the IDtags, we traced variation in the mixed population to eight wild strains representing both mating types from both the Louisiana and Caribbean populations (Fig. 4 and S4). The dominance of the genomic content originating from four strains (3223, 4715, P4452, and 8850), in distinct combinations, was particularly striking (Fig. 4 and S4). Content from 8874, 4713, 3200, and 3199 was also observed (Fig. S4).

### Experimental selection reveals dis-integration of GR and reproduction in *N. crassa*

#### Both growth and reproduction increased in response to spore selection

**Fig. 5:**
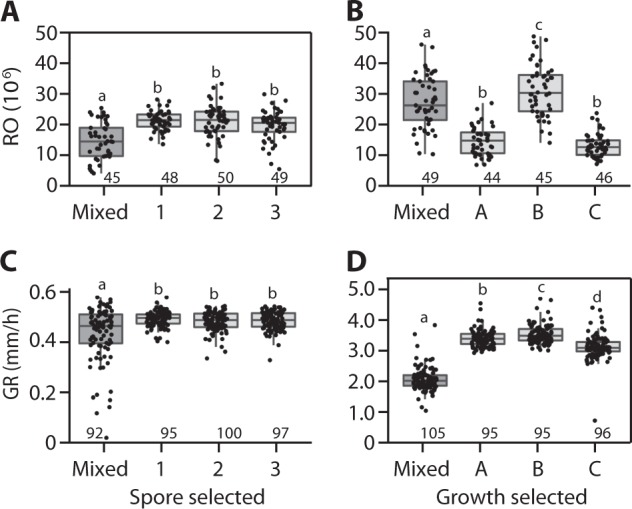
Phenotypic responses in populations selected for either reproductive output or growth rate (**a**, **d**, respectively) and responses in the non-selected traits (**b**, **c**). The mixed population serves as the non-selected ancestral control. Points are means for strains isolated from the mixed population and selected lines. Each strain was measured in triplicate. The number of strains per line is given above the *x*-axis. Results analyzed via analysis of variance with Tukey’s HSD were used for comparisons among lines. Populations sharing the same letter are not significantly different

Spore-selected replicate lines (1, 2, and 3) produced approximately 40% more spores than the mixed ancestral population, increasing from an average of approximately 14.7 to 21 million spores per strain (Fig. 5a; LSMcontrast *F*_1_ = 51.91, *p* < 0.0001). RO varied among strains within lines (*F*_188_ = 2.66, *p* < 0.0001) and between lines (*F*_3 _= 18.52, *p* < 0.0001). No effect of batch was observed (*F*_1 _= 0.83, *p* = 0.36) and correcting for clones in the mixed population did not qualitatively affect these results.

RO did not trade off with GR in the spore selection lines. Rather, increased RO was accompanied by increased GR in the spore-selected lines relative to the mixed ancestor (Fig. 5c; LSMcontrast *F*_1_ = 59.77, *p* < 0.0001). GR varied among lines overall (*F*_3_ = 20.43, *p* < 0.0001) driven by increased GR in all three selected lines relative to the mixed ancestor (Tukey’s HSD, *p* *≤* 0.05). Variation between lines (above) and among strains (*F*_380_ = 8.06, *p* < 0.0001) was significant (batch *F*_3 = _2.87, *p* = 0.04).

Genomic content in the spore-selected lines originated exclusively from the four wild strains that dominated the mixed population (3223, 4715, P4452, and 8850), including both high spore-producing and low spore-producing wild strains (4715 and 8850, respectively; Fig. 4). One clone, of mating type *mat-A*, rose to high frequency in all three lines (Figs. 3, 4). This clone was not the most frequent clone sampled in our survey of the mixed population (Fig. 3). The high frequency of this clone in each line explains similarities among lines for GR and RO (Fig. 5a, c). Also present in each spore-selected line were strains of the opposite mating type (*mat-a*), other genotypes sampled in the mixed population, and clones neither sampled from other spore lines nor the mixed population. The latter finding emphasizes that the mixed population harbored more diversity than sampled in our survey of 94 random strains.

#### Growth selection reveals that the growth–reproduction relationship is not fixed

Growth-selected lines (lines A, B, and C) grew approximately 33% faster than average in the mixed population (Fig. 5d; LSMcontrast *F*_1_ = 994.87, *p* < 0.0001). GR varied among lines (*F*_3 _= 355.02, *p* < 0.0001) and among strains within lines (*F*_386_ = 1.96, *p* < 0.0001). No effect of batch was observed (*F*_3_ = 1.53, *p* = 0.21). Growth-selected lines produced an average of approximately seven million fewer spores (average 19,596,216 per line) than the mixed population (27,099,255; LSMcontrast *F*_1_ = 46.52 *p* < 0.0001) suggesting a trade-off in performance. However, this average result is driven by significant variation among lines (*F*_3_ = 83.69 *p* < 0.0001) in which selection for increased GR was accompanied by decreased RO in two lines (lines A and C, *mat-A* and *mat-a*, respectively), but significantly increased RO in line B, the fastest-growing selected line (Fig. 5b; Tukey’s HSD ≤0.05; *mat-A*). Variation among strains and between batches were not significant in this assay (strain: *F*_191_ = 1.20, *p* = 0.08; batch: *F*_1_ = 0.01, *p* = 0.92). Line B also differed from lines A and C by displaying a strong circadian sporulation phenotype in the growth assays, possibly due to genomic content from strain 4712 [38]. Overall, we conclude that increased GR can be achieved without detriment to asexual RO in *Neurospora crassa*.

At the genotype level, the growth-selected clones were strikingly different from the spore and mixed populations (Fig. 4). Genomic content in the growth-selected clones originated from nine wild strains, four of which were not detected in the survey of the mixed population (2229, 4712, 8789, and 8848). None of the growth-selected clones were sampled in our survey of the mixed population, indicating these rose from relatively low frequency. DNA from P4452 and 2229 is found in all selected clones, although from different genomic regions and in unique combinations (with 8789 in line A, with 4712 and 4713 in line B, or with 3223 and 8848 in line C). Wild strains with the highest and lowest GRs (8789 and 4713) both contributed to growth-selected clones.

## Discussion

Trade-offs are a well-known concept in life history evolution theory, where fitness is optimized when increases in one life history trait are associated with decreases in another. Here, we tested the relationship between two such traits in *N. crassa* wild strains and separately in a mixed population using experimental evolution, a powerful approach for investigating relationships between life history traits [39]. We used this approach to select for either RO or GR and evaluated outcomes in the non-selected phenotype. By selecting on replicate lines from a heterogeneous lab-derived population (the mixed population; Fig. 3, S1, and S2), we tested the growth–reproduction relationship across the diversity of allelic and epistatic effects present in the mixed population. We found that GR and asexual RO are highly variable and plastic and are independent traits in *N. crassa* in the environments tested. This independence is in conflict with generally accepted predictions from life history evolution and expectations from genetic, resource, and physiological connectedness.

In the spore and growth lines, the selected phenotypes increased by 40 and 33% relative to the average phenotype in the ancestral mixed population. In spore selection, this response was accompanied, in all three lines, by an increase in GR. However, this apparent positive growth–reproduction relationship based on the phenotypic response is potentially misleading because it may be driven by the high frequency of one particular clone after selection (Fig. 3). This dominant clone was not the most frequent clone in the mixed population and is found with other genotypes in each selected line, making experimental contamination unlikely. Rather, this result may be due to selection for a clone that potentially only coincidently had high GR. Lines 1 and 2 contained at least one clone with higher GR and lower RO than the dominant clone or vice versa. Thus, we cannot confidently conclude that in the SGF environment there is a meaningful positive growth–reproduction relationship.

In contrast, growth selection resulted in the fixation or near fixation of unique clones in each selected line (Fig. 3), where each clone rose from low frequency in the mixed population. All three dominant clones have above average GRs (Fig. 5d), but are associated with either high or low RO (Fig. 5b). These data indicate that growth–reproduction relationships are not fixed in *N. crassa*, at least in the sucrose environment, and thus indicate the absence of strict genetic or physiological constraints between these traits. Additionally, we estimated phenotypic correlations for the 20 wild strains and the mixed population in both environments, and found no significant correlation between growth and reproduction under our experimental conditions (Fig. 2 and S3). Overall, GR and asexual RO appear to be largely independent traits in *N. crassa*.

It has long been recognized that life history traits do not always trade off as predicted and that trade-offs can be environment-dependent [6–8, 40]. Here, we tested for a trade-off between GR and RO in two environments, neither mimicking the natural habitat of *N. crassa*. If measured under natural conditions, or different experimental conditions, it is possible that a trade-off would be observed. While the results of this study indicate that these traits are not constrained to trade off, the relationship between GR and asexual RO in nature remains an open question.

In some cases, the absence of a detectable trade-off is due to genetic architecture, such as low or positive pleiotropy [5], where traits have few shared genes or the underlying genes contribute positively to the two traits. Although growth and reproduction share expression of a large number of genes in *N. crassa* [23], it appears that the genetic relationship between these traits is not suitable to generate a trade-off in the environments tested in our experiment.

Although identifying the specific factors impacting the genotypic diversity realized in our populations is beyond the scope of this study, two patterns are notable. First, the high frequency of hybrids in the mixed population with DNA primarily from just four strains (3223, 4715, 8850, and P4452). This population was produced via random mating among strains; thus, its diversity may reflect reproductive incompatibility, variation in sexual or asexual ROs, and selection imposed by the experimental environment during construction of the mixed population. However, the genomic content of mixed strains and the selected clones all include contributions from the Louisiana and Caribbean populations, between which incompatibilities are expected due to their allopatric history [24]. Second is the difference in genotypes found between environments (Fig. 4). The mixed population was produced using SGF as was used for spore selection; thus, resemblance between these populations is unsurprising (Fig. 4). In contrast, the dominant clones from growth selection rose from levels undetectable in our sample of the mixed population, and primarily contained genomic content from 2229, P4452, 8848, 4712, and 8789. Selection for GR and the change in experimental environment likely explain the difference between the growth-selected clones, and the mixed population and spore-selected population.

Beyond informing our understanding of fungal evolution [17, 19, 22, 41], relationships between growth and reproduction in fungi and the environmental dependencies of these traits are of societal importance. For example, phenotypic plasticity in fungi may be a factor in the emergence of fungal diseases involving host jumps and invasions of new environments [42, 43], two processes that may also involve life history changes [44]. Our data suggest *Neurospora* wild strains may be valuable models for understanding intraspecific variation in plastic responses and their regulation [45]. Additionally, an ability to predict the response of one trait based on another would be of practical value in studies of pathology, ecology, and even human allergens [20, 46]. However, the data from our intraspecific study and also from interspecific studies (e.g., ref. [20]) suggest that this may not be possible, even within a species, without additional studies.

Finally, our findings highlight the challenges faced by researchers who seek meaningful yet tractable proxies of fitness for studies in fungi [41]. Commonly, inferences about fitness in fungi are based on measurement of one or few traits presumed to be positively correlated with fitness, with choice among proxies to be informed by knowledge of correlations between traits and organismal biology [41]. GR and/or spore production are often employed for this purpose. The high plasticity we observed in these traits and the lack of predictable correlation between growth and spore production illustrate how use of an inappropriate fitness proxy will lead to erroneous inferences about biological fitness, especially because for many species ecological knowledge is scarce or completely lacking. For example, if spore production on SGF was to actually reflect biological fitness, but GR is employed as a fitness proxy, one would incorrectly infer that strain 4715 was among the least fit, rather than the most fit strains (Fig. S1). Thus, in the absence of robust knowledge of fitness determinants in a particular system, extrapolating to fitness from a single fitness proxy is inadvisable.

### Data availability

RADseq data are available at the NCBI Sequence Read Archive (accession SRP094745 [https://www.ncbi.nlm.nih.gov/sra]). All other data are included in the Supplementary files.

## Electronic supplementary material

SI Methods

Figure S1

Figure S2

Figure S3

Figure S4

Supplemental Data
