# Supplementary material for: Asexual reproduction and growth rate: independent and plastic life history traits in Neurospora crassa
Source: ISME J. 2018 Nov 9;13(3):780–8. doi: 10.1038/s41396-018-0294-7 (PMC6462030; doi:10.1038/s41396-018-0294-7)
Supplement: Supplementary file 1 — SI Methods [file 41396_2018_294_MOESM1_ESM.pdf]

Asexual reproduction and growth rate: Independent and plastic life history traits in *Neurospora crassa*

Supplemental Information—Methods

Strains
Culture Conditions
Generation of mixed population
Phenotype assays
Experimental selection
Clone correction
Tissue growth and DNA extraction
RAD library preparation and sequencing
Sequence data and Genotyping
References

Strains

Details of the wild strains used in this study.

FGSC [1] (Perkins #)	Ellison ID [2]	Mating type	Population	Origin
2229	JW09/ 10	A	Louisiana	Welsh, LA
8870	D110	A	Louisiana	Franklin, LA
3223	JW22	A	Louisiana	Elizabeth LA
3199	JW70	A	Louisiana	Coon, LA
8874	D114	A	Louisiana	Franklin, LA
8871	D111	a	Louisiana	Franklin, LA
3200	JW59	a	Louisiana	Coon, LA
8876	D116	a	Louisiana	Franklin, LA
P4476	-	a	Louisiana	Franklin, LA
P4452	-	a	Louisiana	Franklin, LA
4716	JW49	A	Caribbean	Haiti
8850	D90	A	Caribbean	Uxmal, Yucatan, Mexico
8787	D27	A	Caribbean	Homestead, FL
8789	D29	A	Caribbean	Homestead, FL
4713	JW45	A	Caribbean	Haiti
4715	JW47	a	Caribbean	Haiti
4712	JW43	a	Caribbean	Haiti
8790	D30	a	Caribbean	Homestead, FL
8845	D85	a	Caribbean	Kabah, Yucatan, Mexico
8848	D88	a	Caribbean	Sayil, Yucatan, Mexico

Culture Conditions

Cultures were grown at 25°C on Vogel's medium and modifications thereof unless noted [3]. Solid media was prepared with 1.5% agar; liquid cultures without agar. Vogel's with sucrose (1%) was used for non-colonial growth (*e.g.* liquid culture, growth in race tubes), for phenotype assays in the sucrose culture environment, and as the "paternal" culture condition for the first generation of crosses for the mixed population. Vogel's SGF [Sorbose (1%), Glucose (0.05%), and Fructose (0.05%)] was used for colonial growth during Spore selection, phenotype assays in the SGF environment, as the "paternal" culture condition to produce spores for the second generation of crosses for the mixed population, and for reviving cultures from frozen. Cultures on SGF were typically inoculated with 100 spores/plate by volume. Low nitrogen content favors the production of protoperithecia ("maternal" reproductive structures). Thus, we used Modified Vogel's medium (0.1% NH_4NO_3 w/v [4]) with SGF for "maternal" cultures during production of the mixed population. Mating type tests were performed on standard crossing medium with sucrose [3].

Medium Component	Source
L-(−)-Sorbose	Sigma 85541
D-Fructose	Aldrich 140929
D-(+)-Glucose monohydrate	Fluka 49159
Micro Agar	Duchefa Biochemie M1002
Sucrose	Sigma S0389

Generation of mixed population

The mixed population was produced via two generations of sexual reproduction among the 20 wild strains (WS). The overall crossing process, illustrated in Figure 1 (main text), was as follows (exceptions below). **1)** Wild strains of mating types A (N=10) and a (N=10), illustrated in Fig. 1 as strains 1-3 and 4-6 respectively, were cultured under "paternal" conditions with sucrose (black oval "Petri dishes") and "maternal" conditions with SGF (dashed oval "Petri dishes"). Growth on SGF is colonial and shown as grey circles. **2)** After five days, asexual spores ("spores" herein, grey circles) from each "paternal" WS culture were suspended in tH_20 (water with 0.01% Tween 20, sterile) in individual microcentrifuge tubes. Spore densities were determined by counting with a microscope and haemocytometer. Spores of like mating type (either *mat* a or *mat* A) were mixed in equal numbers, and used to fertilize the "maternal" cultures of the opposite mating type (5000 spores per plate). This was achieved by spreading the suspended spores over the surface of the "maternal" colonies. **3)** Progeny (ascospores, small black ovals) from the first generation of crosses were washed from the lids of the "maternal" plates 3 weeks after fertilization. All collected ascospores were pooled and spore density estimated from two 10 µl drops of well-mixed suspension. **4)** The ascospores from 3 were then cultured under both "maternal" conditions (5000 ascospores total cultured on 45 plates) and "paternal" conditions (3000 total ascospores on 27 plates) with SGF. Inoculated plates were heat treated at 60°C for 35 minutes to stimulate germination, then grown under standard conditions. **5)** Spores were harvested from the "paternal" plates by suspending the spores in tH_2O and transferring the suspension to a common conical tube. Each "maternal" plate was

then fertilized using 10,000 spores (by volume). **6)** Progeny from generation two were harvested by washing lids and plates with tH_2O. The ascospores (N=5000) were plated and heat shocked on SGF as before, to produce spores for freezing at -80°C [5]. This pool of 3rd generation spores constitutes the "mixed population" and cultures of single spore origin from the population are referred to as "mixed strains". Before use in selection experiments, a sample of the mixed population was revived from frozen (~2500 spores) and cultured under standard conditions on SGF for two weeks.

Steps 1 and 2 of the above process were performed three times as above with the following exceptions. In the first round, spores were mixed in equal proportions except for 3200, 8874, and 8876, which were mixed at 2x to counteract low colony densities on the "maternal" plates, and 2000 spores were used per "maternal" WS plate for fertilization.

To reduce stochasticity, in Step 3, if ascospores were produced but did not "shoot" to the plate lid, 15 perithecia (mature fruiting bodies) were picked to the lid and crushed using sterile insect pins. Liberated spores were suspended in water and collected as if they had been naturally ejected.

On average 13 colonies per "maternal" plate produced fruit bodies, with an average of 50 fruit bodies per colony 8 days post fertilization (~29,250 independent fertilization events). Note: these estimates are conservative as only distinct colonies were counted. Four strains failed to reproduce in the "maternal" role (8787, 8871, 4712, 8848).

Phenotype assays

Growth and Spore strains were each assayed concurrently with mixed strains. The Growth+Mixed and Spore+Mixed assays were performed in batches over a total of 2 weeks each assay. Batches contained approximately equal numbers of selected and mixed strains. All mixed and selected strains were grown on the test medium prior to assay. Wild strains were cultured on sucrose prior to assay, and phenotyped separately. In all assays, spores from each strain were suspended in 300 µl of tH_20, manually counted on a haemocytometer, and diluted to 1000 spores/µl for use as inoculum. Strains were assayed for growth and reproduction in triplicate, and all cultures were randomized among racks and trays before incubation.

Spore production: Each culture was inoculated with 50,000 spores by volume. Cultures were grown in tubes (75x12x0.8-1 mm tubes with cellulose stoppers, VWR 212-0013 and 391-0164 respectively) on 0.9 ml uniformly slanted culture medium in 12 h light/12 h dark conditions and moved to 4°C after one week. Spores were suspended in 2 or 3 ml tH_2O (SGF or sucrose media respectively) by vortexing (15 s). The suspension was filtered through Miracloth (EMD Millipore) to remove large hyphal fragments, and diluted into Casyton (5 µl to 5 ml, or 3 µl to 5 ml if too concentrated) for measurement on a Casy DT cell counter (150 µm capillary). Cells between 4-10 µm were counted in three samples of each diluted suspension and the average reported as the value for the sample. Data available in Supplemental Data file Ncrassa_spore_phenotypes.csv.

Linear growth: Race tubes with 12 ml Vogel's sucrose were used to measure linear growth (25 ml non-pyrogenic serological pipettes Sarstedt 86.1685.001) [3, 6]. The dispensing end of each tube was sealed with Parafilm and the other end stoppered with sterilized cotton after inoculation. Race tubes were inoculated with 50,000 spores per replicate and Growth was marked after 24, 48, and 72 h and growth rates calculated from growth between 48 and 72 h. Tubes were exposed to light after inoculation and when marking growth.

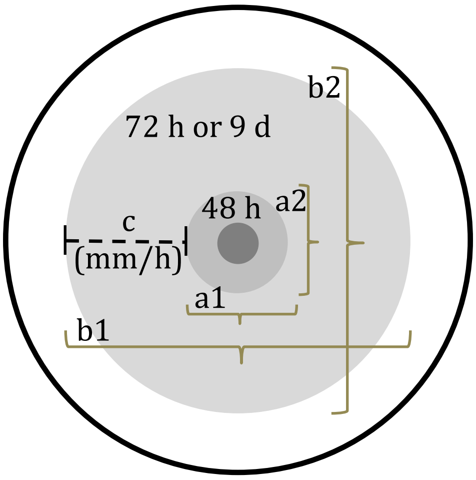

Figure 2. Growth rates were determined from average growth over t hours (24 or 192) as $c = ½*((b1+b2)/2 – (a1+a2)/2))/t$

Radial growth: Vogel's SGF restricts growth, so growth assays on this medium were performed in Petri dishes (55.14.2 mm, 6 vent, VWR 1391-0895) rather than race tubes. Each culture was inoculated with 5,000 spores by volume in the center of the dish and radial growth was marked in perpendicular axes at 24 h intervals for the mixed individuals, and 24 h and 9 days for the wild strains. Radial growth was calculated as described in Figure 1.

Experimental selection

Spores were washed from the plates used to revive the mixed population from frozen (see above; 5ml tH_2O /plate), and diluted to 2spores/µl for spore selection plates and 10 spores/µl for growth selection. Cultures were inoculated with 100 spores/plate or race tube to initiate Spore and Growth selection. Sterile techniques and equipment were used throughout.

Growth selection: Inoculated race tubes (as above) were randomly assigned to replicate A (22 tubes), B (20), or C (20) and grown in the dark at 25°C. After four days, 4 cm of agar containing the terminal growth was harvested from each tube. The terminal growth from all tubes in each line was collected in 1L beakers in 200 ml of tH_2O and then blended with an OBH Nordica Chili hand blender with stainless steel shaft and blade (~ 1 min). The mycelial slurries were transferred to 50 ml conical tubes and centrifuged for 3 min at 2200 rpm and the liquid decanted, leaving a dense slurry. This slurry was used to inoculate the next generation of race tubes (250 µl of slurry per tube). After inoculation, tubes were kept level on the bench overnight (to allow absorption of liquid in the transfer) then put to a dark incubator at 25°C. Tubes were transferred to 4°C after the growth period or when growth reached the end of tube. This process was repeated for 10 generations, with the growth period reduced to 3 days from generation 6. After generation 10, colonized agar fragments (~1mm^2) were isolated from each slurry and used to establish 100 independent cultures from each replicate line (Growth strains).

Spore selection: Inoculated plates were randomly assigned to lines (1, 2, 3), 20 plates per line (SGF), and incubated for one week with 12h light/dark cycles. Spores were

rinsed from each plate using 5 ml tH_2O, filtered through the fiber plug at the top of a serological pipette (to reduce transfer of hyphal fragments), diluted to give 2 spores/µl and spread on fresh plates (100 spores per plate). Spore selection was terminated after 6 generations and the harvested spores frozen at -80°C. Spores were later thawed, diluted, spread on 2% water agar plates, and allowed to germinate overnight. Germinated spores (100 per line) were picked establish the Spore strains.

Clone correction

Clone correction is a curation of the data where potentially redundant data (based on shared genotypes) is removed to reduce bias. This approach is frequently used in population genetics analyses, for example. RADseq results showed that the mixed population as well as the selected lines contain clones, which can affect the outcome of our results. Thus, some analyses were performed using clone correction as follows.

Clone correction was performed on the mixed population, where the main trait of interest is the variation in phenotypic data caused by genetic variation. Clone correction, was performed in two ways. First, in the comparison among strains within the mixed population, we added clone identity as a random factor to the linear model. Second, to estimate the correlation between reproductive output and growth rate, a mean value per trait for each clone was calculated, and used in the Pearson Product Moment Correlations and Spearman's ρ tests. Similarly, to compare the mean response per selection line for growth rate or spore production, relative to the mixed population, these mean values were used. A linear model using the mean value for each clone in the mixed population and the mean value per strain in the selection lines were used as a data points.

For the results from our selection lines, we did not perform clone correction. These lines were started from thousands of spores from the mixed population followed by successive rounds of selection for fast growth or high spore production. We used either asexual spores or fungal mycelium to asexually initiate each generation to assess selection on growth rate or spore production. Each clonal and non-clonal individual contributes to the mean value of those lines' population level trait and any bias from clones in the data actually reflects the mean phenotype of the line after selection. Therefore, we do not think clone correction is appropriate in this study for the selection lines.

Tissue growth and DNA extraction

Each strain was grown in 3 ml liquid cultures at room temperature with shaking. Resulting hyphae was harvested using sterile forceps, excess culture media removed with tissue paper, and the hyphae frozen at -80°C. Approximately 20 mg of hyphae per sample was used for DNA extraction using the ZR-96 Fungal/Bacterial DNA Kit (Zymo Research, Irvine, CA, USA) as directed unless stated. Samples were disrupted 3 times for one minute each at 25 beats per second using a Qiagen TissueLyser II (a fourth round for 1.5 minutes was used if disruption appeared inadequate). After extraction, DNA was eluted from the columns in two steps (40 µl and 35 µl respectively) and stored at 4°C. DNA concentrations were determined using a Qubit 2.0 (Invitrogen) and dsDNA broad range and high sensitivity kits (library preparation) as appropriate.

RAD library preparation and sequencing
Restriction site associated DNA libraries were prepared as previously described [7, 8] unless noted. Briefly, 1 µg of DNA per sample was individually digested using 10 units of *Pst*I (6 bp restriction site; New England Biolabs) at 37°C for 3 h, followed by enzyme deactivation at 80°C for 20 min. The high salt concentration of NEBuffer 3.1 is unfavorable for T4 DNA ligase activity in the next step. To reduce the impact of salts on adapter ligation, 500 ng of digested DNA from each sample was diluted in water 1:2. Adapters with 6 nucleotide barcodes were then ligated to each diluted sample. Adapter sequences are modified from Etter *et al.* [9]. Adapter P1 top 5'ACACTCTTTCCCTACACGACGCTCTTCCGATCTXXXXXXTGC*A and P1 bottom 5'/5Phos/YYYYYAGATCGGAAGAGCGTCGTGTAGGGAAAGAGTGT (*phosphorothioate bond, Xs indicate the position of the barcode). The uniquely barcoded individual samples were pooled (maximum 48 individuals per pool) and the libraries fragmented in a Bioruptor Plus UCD-300 (Diagenode) using the high setting and 13-16 cycles of 30 s on, 60 s off. After ligation of the second adapter (PE-P2 [9]) the libraries were amplified (12 cycles) using Phusion High-Fidelity DNA polymerase (ThermoFisher Scientific) and primers long P1 5'AATGATACGGCGACCACCGAGATCTACACTCTTTCCCTACACGACGCTCTTCCGATC*T and short P2 5'CAAGCAGAAGACGGCATACG*A. Purification of the amplified libraries was performed using Agencourt AMPure beads (1:1 ratio). Libraries with non-overlapping barcodes were combined for sequencing at the SNP&SEQ Technology Platform (Uppsala, Sweden) on an Illumina HiSeq2500 (125 bp reads, v4 sequencing chemistry).

Sequence data and Genotyping
Sequence data were processed using the process_radtags module of Stacks v1.34 (http://catchenlab.life.illinois.edu/stacks/) [10] to demultiplex and quality control the data. Reads with uncalled bases (-c) or low quality scores (-q) were removed, and reads with single errors in the barcodes were rescued (-r, barcodes differed by at least 2 bases). Reads were aligned to the *N. crassa* OR74A NC12 reference genome [11, 12] using GMAP v2015-11-20 [13]) to output reads with only 1 path (-n 1, -Q), using a minimum coverage 95% for alignment, a maximum of 5 mismatches (-m 5), an indel penalty of 2 (-I 2), and a kmer length of 15 (-k 15). The resulting sequence data is available from the NCBI Sequence Read Archive (accession SRP094745; https://www.ncbi.nlm.nih.gov/sra). Variant identification and genotyping was performed using the ref_map module of Stacks allowing up to 3 mismatches between loci when building the catalog (-n 3) and requiring a minimum of 10 identical raw reads to create a stack. Genotypes for 468 strains/individuals were used in subsequent analyses as follows: wild strains n=20; individuals from the ancestral mixed population (strains with ANC_# IDs) n=94; Spore_1 n=95; Spore_2 n=92; Spore_3 n=96; Growth_A n=23; Growth_B n=24, Growth_C n=24.

Identical genotypes (putative clones): To identify unique genome wide haplotypes across different genetic distance thresholds, variant data for more than 12,000 RADtags (loci) genotyped in all individuals in the full study was exported from Stacks in Genepop version 4.1.3 format and analyzed using the Assign Clones function in GenoDive v2.0b27 (Infinite Allele Model, missing data not counted [14]).

IDtags and frequencies: To trace genetic variation from the wild strains to individuals from the lab generated mixed population we used a custom script (match_genotypes.py

written by Jesper Svedberg, Uppsala University; below) that identified strain specific variants among the 20 wild strains and "called" the strain of origin in the mixed individuals. Only RADtags genotyped in all 20 wild strains and without ambiguous base calls were used for this purpose. The data were then used to calculate the frequencies of alleles from each wild strain in the mixed and selected populations. Only RADtags scored in 98% of all sequenced individuals, with 10 or fewer SNPS per tag, and without ambiguous base calls at polymorphic sites genotypes were used for this purpose. Two individuals were excluded due to excessive missing data (spore_1_20, and spore_1_25; N=466). To calculate allele frequencies and haplotypes for all variant positions, we assumed that loci between two IDtags from the same wild strain were also derived from that wild strain, which can be expected from the low number of recombination events. To calculate the variants located between IDtags from different wild strains the mid-point (physical distance) was used as the border to assign alleles.

match_genotypes.py

```
# parent names as header in first row of parent file and likewise for offspring file, tab delimited text files.
# -*- coding: utf-8 -*-
# "parent" refers to wild strains, "offspring" refers to individuals from the mixed population and selected lines.
"""
Created on Fri Aug 14 13:53:00 2015

@author: Jesper Svedberg, Uppsala University
"""

import sys

if len(sys.argv) == 1:
    print "Usage: match_genotypes.py parent_file offspring_file"
    print "Output to terminal."
    sys.exit()
else:
    parfile = sys.argv[1]
    offfile = sys.argv[2]

# Reads files
parlines = open(parfile, "rU").readlines()
offlines = open(offfile, "rU").readlines()

# Extracts and remove table headers
parnames = parlines.pop(0).split()
offnames = offlines.pop(0).split()


typetab = []

# Reads parent table and picks out unique genotypes
for line in parlines:
    typelist = line.split()

    unique = [x for x in typelist if typelist.count(x) == 1]

    ulist = []
    for val in unique:
        ulist.append([parnames[typelist.index(val)], val])

    typetab.append(ulist)

offtab = []

# Reads each cell in the offspring table and checks if it matches one of the
# unique sequences at that locus. If so, it adds the name of the parent
```

```
# strain to an output table
for oc, line in enumerate(offlines):
    linetab = line.split()
    offline = []
    for entry in linetab:
        offline.append("0")
        for typeval in typetab[oc]:
            if entry == typeval[1]:
                offline[-1] = typeval[0]
    offtab.append(offline)


'''
for line in typetab:
    print line
'''

# Prints output table to the terminal
print "\t".join(offnames)

for line in offtab:
    print "\t".join(line)
```

References

1. McCluskey K, Wiest A, Plamann M. The Fungal Genetics Stock Center: a repository for 50 years of fungal genetics research. J Biosciences 2010; 35: 119-126.
2. Ellison CE, Hall C, Kowbel D, Welch J, Brem RB, Glass NL, *et al.* Population genomics and local adaptation in wild isolates of a model microbial eukaryote. P Natl Acad Sci USA 2011; 108: 2831-2836.
3. Davis RH, de Serres FJ. Genetic and microbiological research techniques for Neurospora crassa. Methods in Enzymology. Volume 17, Part A. Academic Press1970, pp. 79-143.
4. Russo VEA, Sommer T, Chambers JAA. A modified Vogel's medium for crossings, mating-type tests and the isolation of female-sterile mutants of *Neurospora crassa*. Neurospora Newsletter 1985; 32: 10-11.
5. McCluskey K, Wiest A, Walker SA. The effect of repeated freeze–thaw cycles on cryopreserved *Neurospora crassa* samples. Fungal Genet Newsl 2006; 53: 37.
6. White B, Woodward D. A simple method for making disposable race tubes. Fungal Genet Newsl 1995; 42: 79.
7. Amores A, Catchen J, Ferrara A, Fontenot Q, Postlethwait JH. Genome evolution and meiotic maps by massively parallel DNA sequencing: Spotted Gar, an outgroup for the teleost genome duplication. Genetics 2011; 188: 799-808.
8. Baird NA, Etter PD, Atwood TS, Currey MC, Shiver AL, Lewis ZA, et al. Rapid SNP discovery and genetic mapping using sequenced RAD markers. PloS one 2008; 3: e3376.
9. Etter PD, Preston JL, Bassham S, Cresko WA, Johnson EA. Local *de novo* assembly of RAD paired-end contigs using short sequencing reads. PloS one 2011; 6: e18561.
10. Catchen J, Hohenlohe PA, Bassham S, Amores A, Cresko WA. Stacks: an analysis tool set for population genomics. Mol Ecol 2013; 22: 3124-3140.
11. Galagan JE, Calvo SE, Borkovich KA, Selker EU, Read ND, Jaffe D, *et al.* The genome sequence of the filamentous fungus *Neurospora crassa*. Nature 2003; 422: 859-868.
12. Stajich JE, Harris T, Brunk BP, Brestelli J, Fischer S, Harb OS, *et al.* FungiDB: an integrated functional genomics database for fungi. Nucleic Acids Res 2012; 40: D675-D681.
13. Wu TD, Watanabe CK. GMAP: a genomic mapping and alignment program for mRNA and EST sequences. Bioinformatics 2005; 21: 1859-1875.
14. Meirmans PG, Van Tienderen PH. GENOTYPE and GENODIVE: two programs for the analysis of genetic diversity of asexual organisms. Mol Ecol Notes 2004; 4: 792-794.
