# Supplementary material for: Asexual reproduction and growth rate: independent and plastic life history traits in Neurospora crassa
Source: ISME J. 2018 Nov 9;13(3):780–8. doi: 10.1038/s41396-018-0294-7 (PMC6462030; doi:10.1038/s41396-018-0294-7)
Supplement: Supplementary file 2 — Figure S1 [file 41396_2018_294_MOESM2_ESM.pdf]

Supplemental figure 1

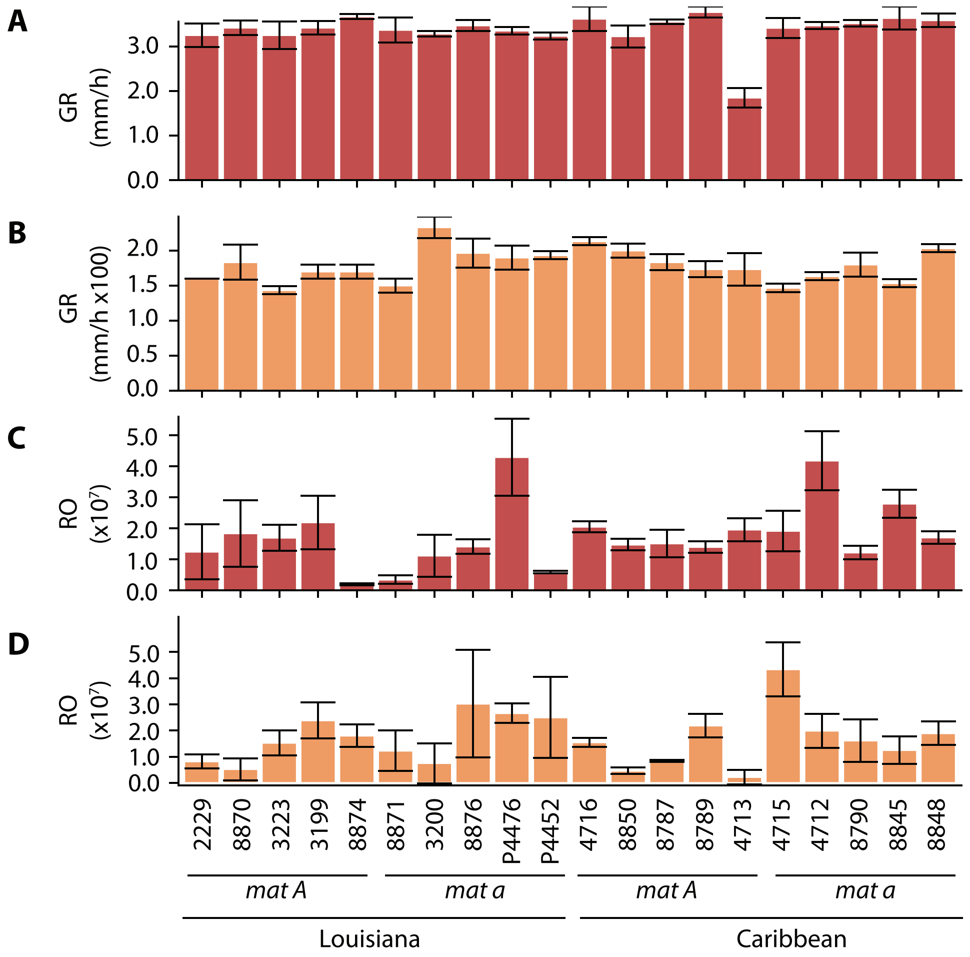

Variation in growth rate (GR) and reproductive output (RO) for 20 wild strains of *Neurospora crassa* on sucrose (red; A,C) and SGF (orange; B,D).Note the that values in B are mm/h x 100. Values are means and error bars are plus and minus one standard deviation.
