# Supplementary material for: Asexual reproduction and growth rate: independent and plastic life history traits in Neurospora crassa
Source: ISME J. 2018 Nov 9;13(3):780–8. doi: 10.1038/s41396-018-0294-7 (PMC6462030; doi:10.1038/s41396-018-0294-7)
Supplement: Supplementary file 3 — Figure S2 [file 41396_2018_294_MOESM3_ESM.pdf]

Supplemental figure 2

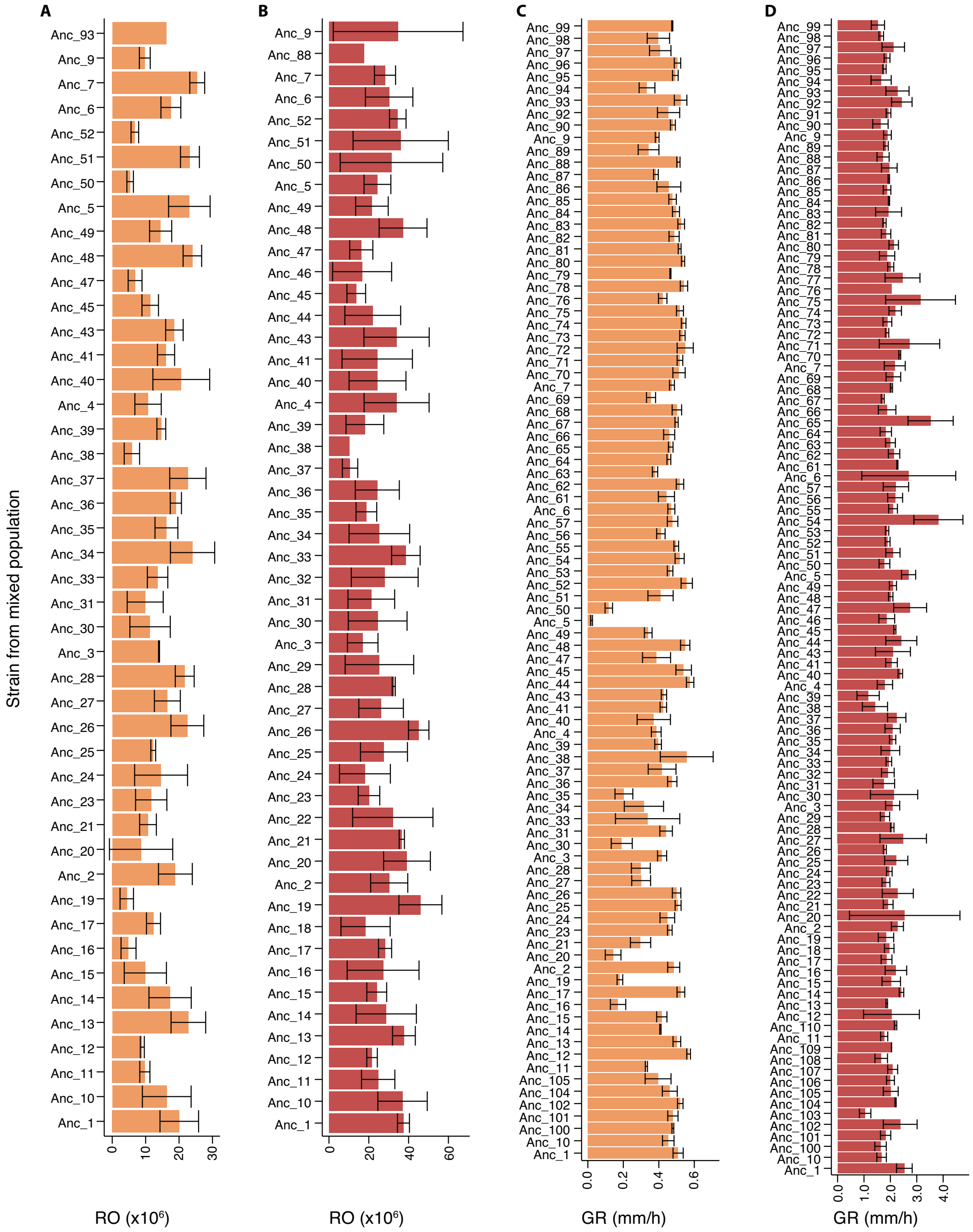

Variation in growth rate (GR) and reproductive output (RO) for strains from the mixed population of *Neurospora crassa* on SGF (orange; A,C) and sucrose (red; B,D). Values are means and error bars are plus and minus one standard deviation.
