# Supplementary material for: Asexual reproduction and growth rate: independent and plastic life history traits in Neurospora crassa
Source: ISME J. 2018 Nov 9;13(3):780–8. doi: 10.1038/s41396-018-0294-7 (PMC6462030; doi:10.1038/s41396-018-0294-7)
Supplement: Supplementary file 4 — Figure S3 [file 41396_2018_294_MOESM4_ESM.pdf]

Supplemental figure 3

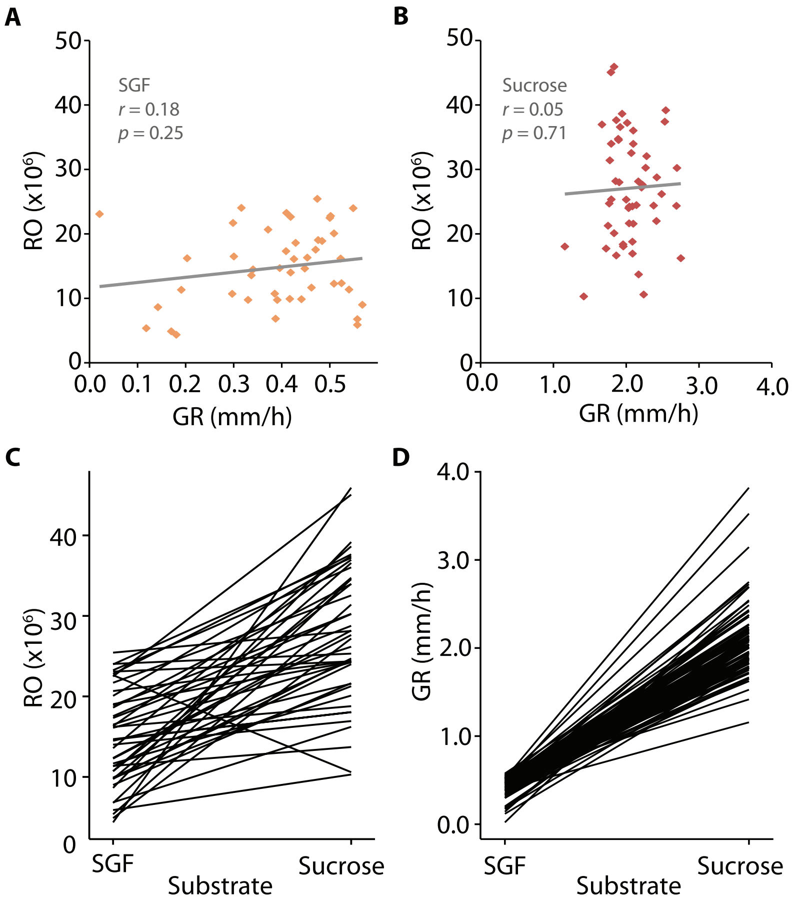


Growth rate (GR) and reproductive output (RO) in *Neurospora crassa* mixed strains. These traits are not correlated either when grown on SGF (orange; A) or sucrose (red; B) and are highly plastic (C, D).
