# Supplementary material for: Asexual reproduction and growth rate: independent and plastic life history traits in Neurospora crassa
Source: ISME J. 2018 Nov 9;13(3):780–8. doi: 10.1038/s41396-018-0294-7 (PMC6462030; doi:10.1038/s41396-018-0294-7)
Supplement: Supplementary file 5 — Figure S4 [file 41396_2018_294_MOESM5_ESM.pdf]

Supplemental figure 4

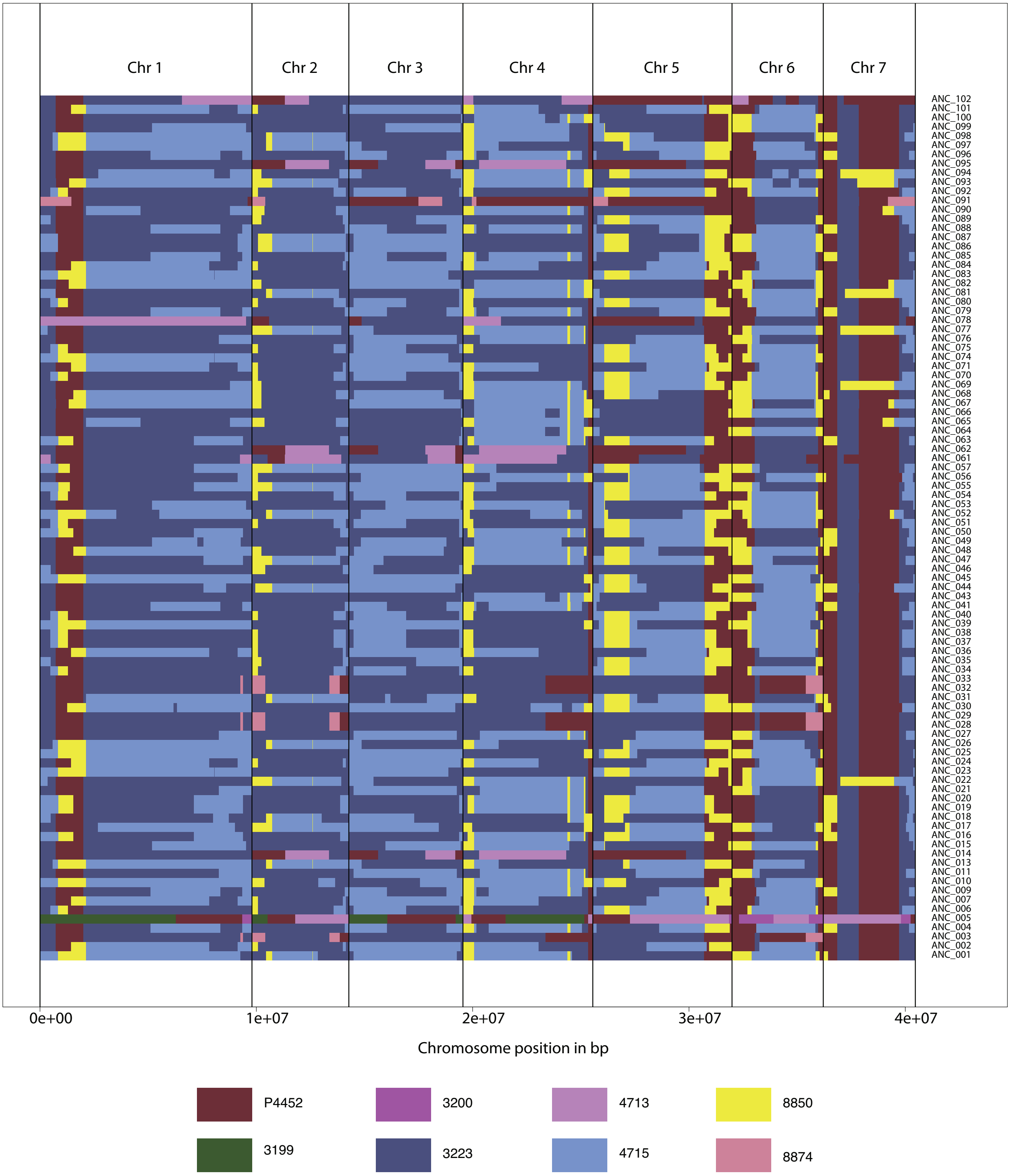

Genome wide plot of the wild strain origins of genomic content in each sequenced mixed strain based on IDtags.
