# Supplementary material for: Asexual reproduction and growth rate: independent and plastic life history traits in Neurospora crassa
Source: ISME J. 2018 Nov 9;13(3):780–8. doi: 10.1038/s41396-018-0294-7 (PMC6462030; doi:10.1038/s41396-018-0294-7)
Supplement: Supplementary file 6 — Supplemental Data [file 41396_2018_294_MOESM6_ESM.gz › Supplemental_data_Anderson_et_al/README_Anderson_et_al.rtf]

Asexual reproduction and growth rate: Independent and plastic life history traits in Neurospora crassaAnderson JL, Nieuwenhuis B, and Johannesson HNote:—The mixed strains and population are referred to as ancestor/ancestral/ANC throughout.—The wild strains are referred to as parent/parental throughout.Ncrassa_spore_phenotypes.csv	Ncrassa_wildstrains_spore_phenotypes.csv	Raw data from spore phenotype assays and associated estimated spore counts for the original cultures.	Output from CASY DT with the following standard values:	Total counts/ml = 1	Capillary Diameter = 150 µm	Left cursor = 4 µm (lower bound of spore size range)	Right cursor = 10 µm (upper bound of spore size range)	Serial Number DT identifier = DT-2ID-1109	Measuring cycles per sample = 3	Sample volume = 212.62 (µl)	Output from the CASY DT included Counts and Counts/ml for each size class (see below). To determine the 	number of spores of each size class produced by the strain (the number of spores in each culture tube) 		calculations were required. These are detailed below.	Column ID— group: spore = Spore selection; growth = Growth selection; ancestor = mixed  population; parental = wild strain— batch: Growth+mixed 1.1, 1.2; Spore+mixed 2.1 and 2.2; parental— assayed_on: Vogel’s medium with sucrose or SGF— line: ANC, Growth A, B, C, or Spore 1, 2, 3— strain: line_# or strain ID— spore_suspension_volume: Volume (µl) of water with 0.01% Tween 20 used to harvest spores— sample_volume: Volume (µl) of harvested spores taken from sample (above) to dilute for measurement.— casyton_volume: Volume (µl) of Casyton solution used to dilute sample.— dilution_factor: of sample_volume to casyton_volume— Counts: total number of cells counted in 3 measuring cycles per sample.— Counts/ml < CL: Counts per ml of measured sample smaller than cursor left (4 µm)— Counts/ml >CL <CR: Counts per ml of measure sample in target cell size range (4-10 µm), or as noted by the machine, between the left and right cursors.— Counts/ml > CR: as above for cells larger than 10 µm	— Total_Counts/ml: = Counts/ml < CL +  Counts/ml >CL <CR + Counts/ml > CR— number < CL: Number of cells of this size in the actual counted sample = (Counts/ml < CL / Total_Counts/ml) x Counts.	— number >CL < CR : as above but for this size class.	— number > CR: as above but for this size class. — conc/ul <CL_per_measured_sample: The concentration of cells of this size class per measurement cycle of the cell 	suspension = (number < CL / 3 measuring cycles)/ 212.62 µl. See sample volume above.	— conc/ul >cL<Cr_per_measured_sample: as above but for this size class.	— conc/ul >cr_per_measured_sample: as above but for this size class.— original number <CL: Estimated number of spores in the original culture based on dilutions and spore 		counts in the sample. = conc/ul <CL_per_measured_sample x dilution_factor x spore_suspension_volume— original # mid range: As above for cells in the target cell size range.— original number > CR: as above for cells larger than 10 µm.Ncrassa_linear_growth_phenotypes.csvNcrassa_wildstrains_linear_phenotypes.csv	Raw data from linear growth phenotype assays and associated calculated growth rates.	Column ID— batch: Growth+mixed 1.1, 1.2, 1.3, or 1.4; parental— date inoculated: month/date/year— time inoculated: 24 hour— strain: line_# or strain ID— mm 24-48: growth (mm) in the 24-48 h time interval— mm 48-72: growth (mm) in the 48-72 h time interval— caution_1: 1 = small colony observed upstream of 48 h mark that might impact growth. 0 = no caution. Not in the wild strain dataset.—caution_2: 1 = 24-48 mark suspect due to size—potential spread of inoculum up tube. 0 = no caution. Not in the wild strain dataset.— score_1: actual hours post inoculation for end of first scored growth period— score_2: actual hours post inoculation for end of second scored growth period— growth_24-48: growth rates for the actual elapsed time during the time interval— growth_48-72: growth rate for the actual elapsed time during the time intervalNcrassa_radial_growth_phenotypes.csvNcrassa_wildstrains_radial_phenotypes.csv	Raw data from radial growth phenotype assays and associated calculated growth rates. See Supplemental 	Methods for more information on measurements and calculations.		Column ID— batch: Spore+mixed 2.1, 2.2, 2.3, or 2.4; parental— date: month/date/year inoculated— time: 24 hour— strain: line_# or strain ID— measure1_24h: mm from one edge of the culture to the opposite edge after 24 h — measure1_48h: mm from one edge of the culture to the opposite edge after 48 h— measure1_216h: mm from one edge of the culture to the opposite edge after 216 h. Wild strain file only.— measure1_72h: mm from one edge of the culture to the opposite edge after 72 h — measure1_72h_edge: If growth had reached the edge of the plate, it is noted as an “e” and was given a 	value of 52 mm for “measure1_72h”. This may be an underestimate of the growth rate for these samples. 	— measure2_24h: as for measure 1 above but from the perpendicular axis— measure2_48h: as for measure 1 above but from the perpendicular axis— measure2_216h: as for measure 1 above but from the perpendicular axis. Wild strain file only.— measure2_72h: as for measure 1 above but from the perpendicular axis 	— measure2_72h_edge: as for measure 1 above but from the perpendicular axis— rate_24-48: radial growth rate in mm/h based on growth in the 24-48 hour interval — rate_48-72: radial growth rate in mm/h based on growth in the 48-72 hour interval— rate_24-216: radial growth rate in mm/h based on growth in the 24-216 hour interval. Wild strain file only.Ncrassa_genepop_clones.txtFile with data for 12,657 SNPs in Genepop format used to identify clones. The locus ID’s, listed before the first “pop” correspond to the “Catalog ID” and the position of the SNP in the sequence of that tag as detailed in “SNPs” in the file Ncrassa_genotypes_and_tags.txt.Ncrassa_cloneIDs.csvClone IDs assigned by GenoDive 2.0b27, 2016-07-21 using Assign Clones with 468 and 12657 loci included, with thresholds 0 and 1000.	Column ID— strain: line_# or strain ID— clone_0— clone_1000Ncrassa_genotypes_and_tags.txt	Data generated by Stacks including information for each RADtag and the haplotype of each strain at each 	locus. 	Column ID— Catalog ID: ID of a locus identified in Stacks, also called RADtag— Chr: Chromosome location of the RADtag based on the N. crassa OR74A NC12 reference genome— BP: base pair position of the tag on the chromosome— Consensus Sequence: The consensus sequence (majority rules) for the RADtag— Num strains: The number of strains genotyped— Num SNPs: The number of polymorphic sites found in the RADtag— SNPs: The position in the sequence where the SNPs are found (starting from 0), followed by the consensus and alternate bases at that position— Num Alleles: The number of alleles identified for the RADtag— Alleles: The observed alleles/haplotypes— The remaining columns are strain IDsNcrassa_full_data.vcf.zipVariant call format file of all RAD data generated by Stacks. This file contains 7 strains that were excluded from analyses (ANC_12, ANC_72, growth_A_60, spore_1_86, spore_2_47, spore_2_52, and spore_2_87) due to known problems during sample preparation and two wild strain technical replicates (“name_copy”). These samples should be excluded from further study and their sequences have not been deposited to SRA.Ncrassa_IDtags.csv	This file contains the full dataset (8555 RADtags) used to identify 6423 IDtags and the resulting IDtag data	Column ID— Catalog ID: ID of a locus identified in Stacks, also called RADtag— Chr: Chromosome location of the RADtag based on the N. crassa OR74A NC12 reference genome— BP: base pair position of the tag on the chromosome— Consensus Sequence: The consensus sequence (majority rules) for the RADtag— SNPs: The position in the sequence where the SNPs are found (starting from 0), followed by the consensus and alternate bases at that position— The next columns give the haplotype data for each strain. Followed by the IDtag result for each strain. 0 = no IDtag called at that site for that strain.Ncrassa_meansPerStrain_withCloneCorrectedMixedPop.csv	This file contains the mean values for the phenotypes of the mixed strains and evolved populations in which the values for the mixed strains are the mean value per clone.	Column ID— population: 1, 2, 3 for spore selected lines; A, B, C for growth selected lines and Anc for mixed population— isolate: strain number for the evolved lines and for the mixed population the clone_ID from Ncrassa_IDtags.csv— clone_0: clone_ID for each of the strains — meanSGF: mean growth rate measured on SGF per strain or clone; for the evolved lines the mean of the replicates per strain and for the mixed strains the mean per clone— meanSucr: mean growth rate measured on sucrose medium per strain or clone; for the evolved lines the mean of the replicates per strain and for the mixed strains the mean per clone— meanSporeSucr: mean spore production measured on sucrose medium per strain or clone; for the evolved lines the mean of the replicates per strain and for the mixed strains the mean per clone— meanSporeSGF: mean spore production measured on SGF per strain or clone; for the evolved lines the mean of the replicates per strain and for the mixed strains the mean per clone
